# Unlocking the transcriptomic potential of formalin-fixed paraffin embedded clinical tissues: comparison of gene expression profiling approaches

**DOI:** 10.1186/s12859-020-3365-5

**Published:** 2020-01-28

**Authors:** Arran K. Turnbull, Cigdem Selli, Carlos Martinez-Perez, Anu Fernando, Lorna Renshaw, Jane Keys, Jonine D. Figueroa, Xiaping He, Maki Tanioka, Alison F. Munro, Lee Murphy, Angie Fawkes, Richard Clark, Audrey Coutts, Charles M. Perou, Lisa A. Carey, J. Michael Dixon, Andrew H. Sims

**Affiliations:** 1 Applied Bioinformatics of Cancer, Cancer Research UK Edinburgh Centre, MRC Institute of Genetics and Molecular Medicine, Edinburgh, UK; 2 Edinburgh Breast Unit, Western General Hospital, Edinburgh, UK; 3 Department of Pharmacology, Faculty of Pharmacy, Ege University, 35040 Izmir, Turkey; 4 Usher Institute of Population Health Sciences and Informatics, Old Medical School, Teviot Place, Edinburgh, UK; 5 Lineberger Comprehensive Cancer Center, University of North Carolina, Chapel Hill, NC USA; 6 Host and Tumour Profiling Unit, Cancer Research UK Edinburgh Centre, MRC Institute of Genetics and Molecular Medicine, University of Edinburgh, Edinburgh, UK; 7 Edinburgh Clinical Research Facility, Western General Hospital, Edinburgh, UK

**Keywords:** FFPE, Fresh-frozen, Gene expression, Microarray, Sequencing, Transcriptomics

## Abstract

**Background:**

High-throughput transcriptomics has matured into a very well established and widely utilised research tool over the last two decades. Clinical datasets generated on a range of different platforms continue to be deposited in public repositories provide an ever-growing, valuable resource for reanalysis. Cost and tissue availability normally preclude processing samples across multiple technologies, making it challenging to directly evaluate performance and whether data from different platforms can be reliably compared or integrated.

**Methods:**

This study describes our experiences of nine new and established mRNA profiling techniques including Lexogen QuantSeq, Qiagen QiaSeq, BioSpyder TempO-Seq, Ion AmpliSeq, Nanostring, Affymetrix Clariom S or U133A, Illumina BeadChip and RNA-seq of formalin-fixed paraffin embedded (FFPE) and fresh frozen (FF) sequential patient-matched breast tumour samples.

**Results:**

The number of genes represented and reliability varied between the platforms, but overall all methods provided data which were largely comparable. Crucially we found that it is possible to integrate data for combined analyses across FFPE/FF and platforms using established batch correction methods as required to increase cohort sizes. However, some platforms appear to be better suited to FFPE samples, particularly archival material.

**Conclusions:**

Overall, we illustrate that technology selection is a balance between required resolution, sample quality, availability and cost.

## Background

Since their inception microarrays have been adopted as a major tool for the study of clinical samples to improve our understanding of diseases, development of molecular subtyping and prognostic signatures for clinical decision-making [1]. A crucial consideration for many clinical studies is whether new data generated can be directly compared or integrated with pre-existing datasets for robust classification and response prediction.

RNA sequencing (RNAseq) has somewhat supplanted microarrays for transcriptome analysis. However, in translational research when the focus is often restricted to identifying differentially expressed genes and pathways, rather than detecting specific isoforms and splice variants, decisions on which platform to use are often based upon cost, rather than resolution, particularly if this means more samples can be examined to maximise statistical power for a fixed budget. Indeed, RNAseq is not without its limitations, Robert and Watson recently demonstrated that RNAseq is unable to accurately measure expression of hundreds of genes in the human genome [2].

Many high-throughput profiling studies rely on sample availability and cost rather than statistical power [1]. Direct integration of datasets enables meta-analysis and has the potential to improve statistical power and the generalisability of results for robust classification and response prediction. However, non-trivial systematic bias or ‘batch effects’ can occur within and between microarray platforms [3–6]. Contrary to The MicroArray Quality Control guidelines [7], gene expression data can be directly integrated and robust results can be produced from fundamentally different technologies such as Affymetrix GeneChips and Illumina BeadChips [3]. This finding has since been supported by other studies [8, 9].

Early microarray studies involving clinical samples were dependent on relatively large amounts of high quality RNA and thus relied heavily on the availability of fresh frozen (FF) tissue. However, collection and storage of FF tissue is costly and can be logistically prohibitive. Protocols and technologies capable of generating high quality whole-genome transcriptomic data from archival formalin fixed paraffin embedded (FFPE) tissues are in demand [10]. FFPE tissues are available routinely in the clinical setting and can be stored at ambient temperature for many years, allowing easy transportation. A large number of studies have compared matched FF and FFPE samples, with some reporting reduced efficacy or numbers of detected transcripts and batch effects similar to those reported for different profiling technologies (recently reviewed [11]). Most studies conclude that the data can be compared to some extent, subject to certain considerations, accepting that RNA from FFPE samples is often degraded and continues to degrade with age [10]. Whilst earlier microarray technologies performed poorly with degraded RNA, newer kits and platforms have emerged using targeted sequencing such as Ion AmpliSeq Transcriptome and BioSpyder TempO-Seq or 3′ sequencing from Lexogen QuantSeq. Other technologies such as NanoString are promising, but are limited to panels of genes rather than whole genome transcriptome. In this study, a number of gene expression profiling platforms were compared.

## Methods

### Clinical samples

All patients gave informed consent and the study was approved by the local ethics committee (LREC; 2001/8/80 and 2001/8/81). RNA was extracted from primary human breast cancer samples collected over 15 years at the Edinburgh Breast Unit from post-menopausal women with estrogen receptor positive disease, treated with 3-months of neoadjuvant endocrine therapy. Sequential biopsies were taken pre-treatment, early (14-days) on-treatment and at surgery 3–6 months later (late on-treatment) from each patient. Part of the biopsy material collected was snap-frozen in liquid nitrogen and part was fixed in formalin and embedded in paraffin. RNA was extracted from fresh frozen tissue using the Qiagen miRNeasy kit and from 2 × 20 μm FFPE tissue sections using the RNeasy FFPE kit using the manufacturer’s standard protocols for each kit. Agilent RIN values for fresh frozen tissue were > 7 and for FFPE tissue were < 3.

### Transcriptomics

Building upon large scale clinical studies to investigate the effects of endocrine therapy on breast cancer using Affymetrix U133A arrays [12] and Illumina HT12-V4 BeadChips [13], this study, utilised patient-matched sets of samples across a range of transcriptomic technologies: Affymetrix Clariom S, NanoString, Ion AmpliSeq Transcriptome, BioSpyder TempO-seq [14] Lexogen QuantSeq and RNA-seq (Table 1). Microarray samples were processed as directed by the manufacturer’s instructions. Nanostring profiling was performed using nCounter technology as per the manufacturer’s instructions. Sequencing was performed as described: Ion Ampliseq samples were processed using an Ion a PI™ Chip Kit v3 and sequenced using an Ion Proton™ System. QiaSeq samples were sequenced using the NextSeq 500/550 High-Output v2 (150 cycle) Kit on the NextSeq 550 platform. For TempoSeq samples, single read (1x75bp) sequencing was performed using the NextSeq 500/550 High-Output v2 (75 cycle) Kit on the NextSeq 550 platform. For QuantSeq samples were either processed via single read (1x75bp) sequencing performed using the NextSeq 500/550 High-Output v2 (75 cycle) Kit on the NextSeq 550 platform or via Ion a PI™ Chip Kit v3 and sequenced using an Ion Proton™ System. For RNASeq samples the TruSeq Stranded Total RNA Library Prep Kit with Ribo-Zero Gold (Illumina) was used and sequencing was performed on an Illumina HiSeq 2500 using a 2x50bp configuration with an average of 136 million read pairs per sample. All data is publicly available from NCBI GEO (www.ncbi.nlm.nih.gov/geo/) under super-series accession GSE130645.

**Table 1:** Comparison of traditional and new microarray platforms with sequencing approaches

Technology	Technology/Platform	Biochemistry	Approx. Throughput	Max. no. probes/primer pairs	No. of mapped ENSG IDs	Read Depths	Input FFPE RNA (ng)*	Approx. cost per sample (£)**	Success rate of FF samples (n)	Success rate of FFPE samples (n)
3′ RNA sequencing	Lexogen QuantSeq	RNA → RT, oligodT priming from 3′ end, random priming towards 3′ end → amplification and barcoding → sequencing	96 samples per 5 days	55,765	25,610	10 M	500	90	N/A	98% (318)
	QiaSeq UPX 3′ Transcriptome	RNA → RT, oligodT priming for cDNA synthesis →template switching for 2nd strand synthesis priming → fragmentation → end repair addition, adapter ligation → PCR to add indices → sequencing	96 samples per 5 days	42,553	20,000	15 M	10	50	N/A	94% (48)
Specific Targeted Sequencing	BioSpyder TempO-Seq	RNA → annealed 50 bp detector oligos are ligated then amplified and barcoded → sequencing	192 samples per 4 days	19,300	19,300	12 M	20 μm FFPE Section	160	N/A	95% (38)
	Ion Ampliseq Transcriptome	RNA → RT, multiplex PCR → sequence barcoding → emulsion PCR → sequencing of ~ 150 bp targets	96 samples per 5 days	20,802	19,059	8 M	10	160	100% (108)	76% (76)
Targeted Probes	Nanostring	RNA → hybridisation to fluorescent barcoded probes in solution → immobilised in nCounter cartridge → scan	12 samples per day (800 genes)	800	800	N/A	50	250	N/A	100% (12)
Newer Microarray	Affymetrix Clariom S	RNA → cRNA amplification → hybridisation to GeneChip → scan	192 samples per 4 days	211,300	> 20,000	N/A	50	100	100% (3)	100% (8)
Traditional Microarray	Affymetrix U133A		192 per day	250,833	11,827	N/A	50	360	100% (178)	100% (286)
	Illumina BeadChip HT-12 v3 / v4	RNA → RT, amplification, biotinylation (NuGEN WT Ovation kit) → hybridisation to 50 bp probes on chip → scan	96 samples per 1.5 days	47,323	22,571	N/A	1500	195	91% (348)	21% (206)
Full RNA Sequencing	RNA-seq	RNA → fragmentation → RT → barcoded library construction → genome-wide full RNA sequencing	8 samples per 5 days	20,025	18,57s1	136 M paired reads	2000	250–500	100% (52)	100% (87)

*Input RNA reflects quantities used in this study – for input ranges refer to the manufacturer’s guidelines

**Estimated costs (£, UK December 2019) include library preparation and sequencing. Costs can vary by sample numbers and sequencing infrastructure

### Data analysis

Illumina and Affymetrix data were pre-processed and normalised as described previously [3]. NanoString data were generated using the nSolver 3.0 software. Ion AmpliSeq Transcriptome data were generated using the AmpliSeq RNA plugin in the Torrent Suite Software and normalised using RPM (reads assigned per million mapped reads) method. QiaSeq FASTQ files were uploaded to the GeneGlobe Data Analysis Center, an online platform provided by QIAGEN. The primary analysis module for the UPX 3′ Transcriptome Kit was used to generate UMI-based gene expression estimates from the reads for all samples. QuantSeq raw data in .bcl format was transferred from the NextSeq instrument to a Linux system, where demultiplexed FASTQ files were generated using Bcl2fastq2 v2.17.1.14 software provided by Illumina. The lane-splitting feature was disabled to create a single FASTQ file for each library. FASTQ files were then uploaded to the BlueBee genomics platform (https://www.bluebee.com) and read-trimming and alignment was performed using the QuantSeq plugin. TempoSeq FASTQ files were sent to BioCalvis (the manufacturer of BioSpyder), who performed the alignment and then generated the raw (un-normalised) gene counts file using their proprietary software. For RNAseq, alignment was performed using STAR74. Transcript abundance estimates for each sample were performed using Salmon, an expectation-maximization algorithm using the UCSC gene definitions. Raw read counts for all RNAseq samples were normalized to a fixed upper quartile.

All sequence data were aligned to the human reference hg19 genome. For all data, probes or genes were then mapped to Ensembl gene annotations: Affymetrix datasets were mapped using a chip definition file (CDF) [15] and all other datasets were mapped using BioMart. All data were Log2 transformed and filtered for those expressed in 70% of samples using the cluster 3.0 software then quantile normalised using the R/Bioconductor software and packages [16]. Following data integration, correction of systematic bias was performed using ComBat as described previously [3].

## Results

### Performance and cost comparison of platforms for FF and FFPE tissue

**Fig. 1:**
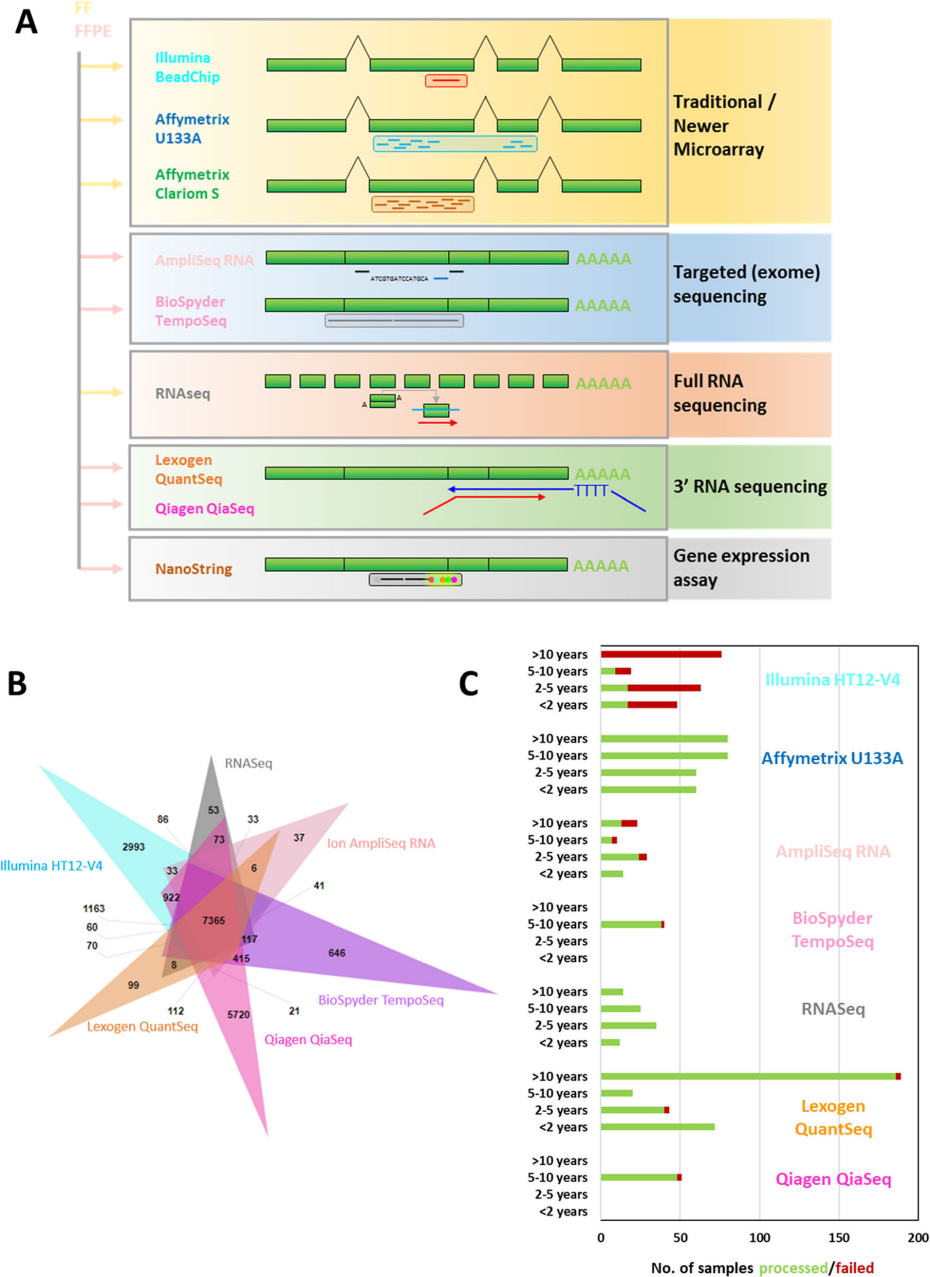
Comparison of gene expression profiling approaches (**a**) Schematic of probe/primer designs for each technology. A table showing which samples were processed on each technology is provided in Additional file 1: Table S1. **b** Number of overlapping Ensembl gene identifiers detected in each dataset (Nanostring and Affymetix U133 were omitted as they do not represent the whole transcriptome and the Clariom S was excluded as only three samples were processed). **c** Summary of FFPE sample processing success rates by sample age using whole-transcriptome platforms

Each of the nine technologies evaluated here have different mRNA input requirements, probe designs (Fig. 1a) and protocols (summarised in Table 1). Although the total number and position of probes/primers/counts varies widely among the transcriptome-wide approaches (Table 1, Fig. 1a), a common set of 7365 Ensembl transcripts were represented across the six whole transcriptome platforms (Fig. 1b). Nanostring and Affymetrix U133 were omitted as they do not represent the whole transcriptome and the Clariom S was excluded as only three samples were processed). RNAseq may have the highest resolution, but also the highest RNA input requirement (100-4000 ng) and it is the most expensive whole transcriptome technology at two to five times the cost of other approaches (Table 1). The NanoString platform could be cost-effective for a small number of genes, but compares poorly on price for large numbers of genes (costed for maximum coverage in a single experiment: 770 genes). The newest and least expensive technologies are Affymetrix Clariom S array with WT Pico kit and Lexogen QuantSeq. Success rate is an important consideration for clinical studies, particularly with before and on-treatment matched samples considered in this study. Looking at the numbers of samples which have failed using different technologies based on the respective manufacturers quality control criteria we found that success rates for generating robust expression profiles from FFPE tissues were excellent (> 95%) for the latest Lexogen QuantSeq, Qiagen Qiaseq, BioSpyder TempO-Seq methods. This is despite the RNA integrity number (RIN) values for fresh frozen tissue normally being above 7, but for FFPE tissues were generally less than 3. However, success rate was moderate for the Ampliseq RNA Transcriptome (83%) and poor for the older Illumina BeadChip (22%). By comparison RNA from FF tissue had a high success rate (91–100%) with several hundred samples processed on the Illumina BeadChip, Affymetrix U133A chips and RNAseq (Table 1). As shown previously [10], older FFPE samples were found to perform very poorly with the more established technologies (Fig. 1c) whereas NanoString, Lexogen QuantSeq and RNA-seq were found to work well with old FFPE tissue-derived RNA.

### Integration of datasets across platforms while preserving biological variability

To evaluate how newer technologies with desirable features such as lower costs or RNA input requirements compared to the more established methodologies, we profiled the same RNA from a subset of samples to directly compare gene expression measurements across the platforms (Additonal file 1: Table S1). These comparisons have two purposes; firstly to determine whether the new technology provides similar quality results to the established method. Secondly, to evaluate whether it will be possible to directly integrate datasets generated on the new platform with existing local or publicly available data from another platform, as we have done previously [3, 4, 6]. Indeed, while it is altruistic to minimise measurement error by using the same platforms, with constantly evolving technologies and lower associated costs this is not often realistic. Therefore, the ability to implement approaches to increase validity across platforms is of great importance.

**Fig. 2:**
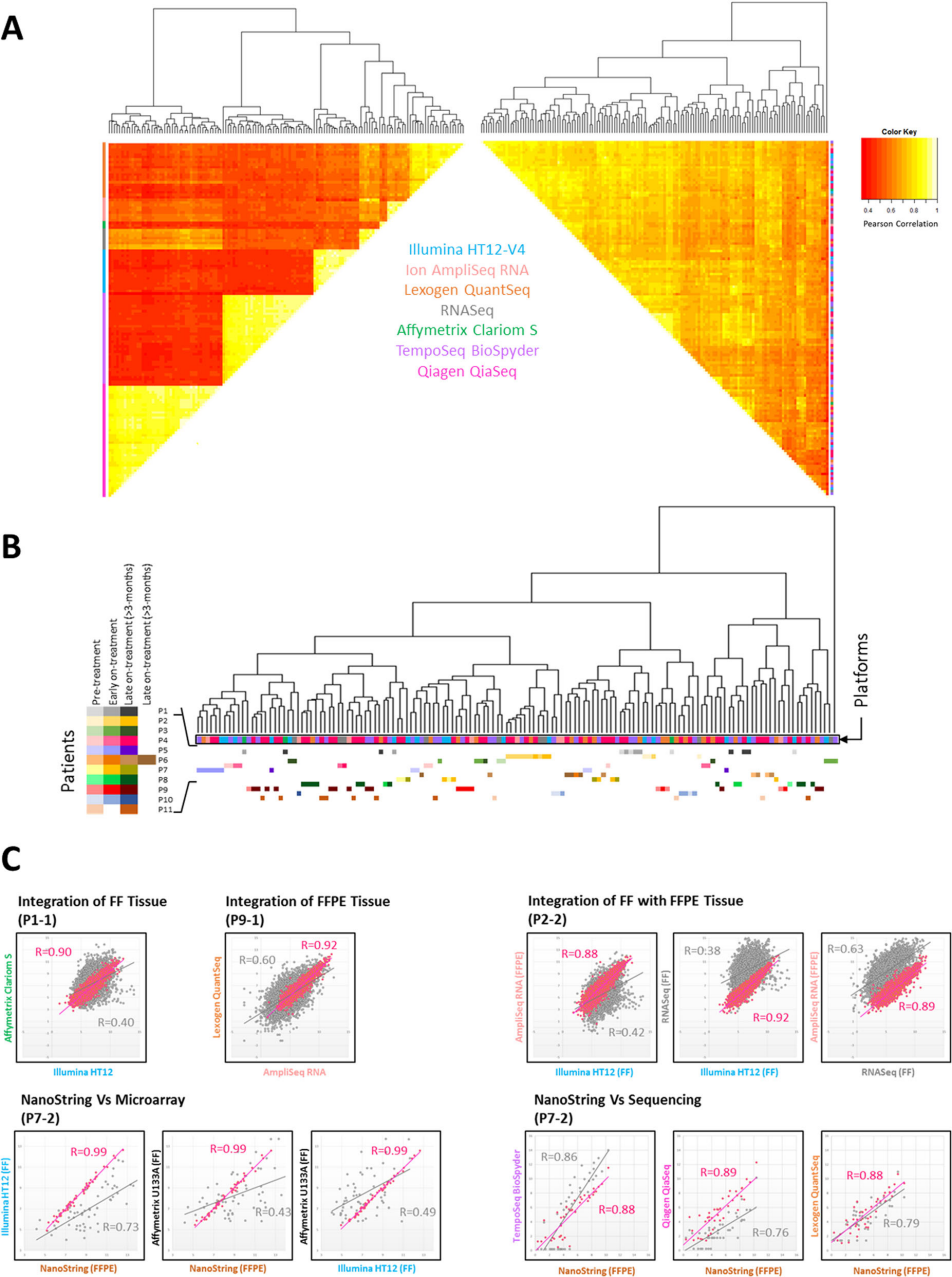
Batch correction allows robust direct integration of transcriptomic data across platforms. **a** Dissimilarity heatmaps based upon Pearson correlations ranging from 0.4 (red) through shades of orange and yellow to 1.0 (white). Left triangle shows the combined dataset of 6844 genes across 7 gene expression platforms. Right triangle shows the same data following batch correction with Combat. Coloured bars below dendrograms denote the platform. **b** Enlargement of the dendrogram to demonstrate that the majority of the same time-point patient samples processed on different platforms cluster together following batch correction. **c** Scatter plots before (grey) and after batch correction (pink) of the same sample, either FF or FFPE processed across different platforms. In each case the Pearson correlations increase substantially following batch correction. Patient samples are denoted − 1 for pre-treatment, − 2 for early on-treatment

Not surprisingly, when all samples were integrated together low correlations (*r* = 0.4–0.6) were observed between pairs of samples processed on different technologies. Hierarchical clustering showed clearly that gene expression values group by technology and technical artefacts, rather than by genuine biology (Fig. 2a, left). Following batch correction using the well-established and highly cited ComBat method [17], correlations were much higher and the majority of ‘paired’ samples clustered together, indicating greater variation between biological samples than between gene expression measurement platforms (Fig. 2a, right). Looking more closely, instances of the same time-point processed on different platforms clustered closely (if not together) and different time points from the same patients showed variation (due to treatment), whilst also often clustering with other time points from the same patient (Fig. 2b), as has been previously shown for sequential patient-matched samples [13]. These results are consistent with our previous results showing a reduction in technical artifacts, without loss of biological variation [3].

Clear batch effects were evident when comparing mRNA extracted from FF samples across Illumina HT12, Ion Ampliseq Transcriptome and Affymetrix Clariom S, with low Pearson correlations (*r* = 0.4–0.58). However standard batch correction approaches such as ComBat [17] minimised technical bias effect and increased correlation to *r* > 0.9 for paired samples. Similar low correlations and improved correlations following batch correction were observed for different technologies with FFPE samples and for comparisons of matched FF and FFPE or for the same sample across different platforms (Fig. 2c). Comparison of measurements of the 56 overlapping genes assayed using NanoString, whole-genome (Illumina HT12) and part-genome (Affymetrix U133A) expression microarrays were also significantly improved following batch correction.

**Fig. 3:**
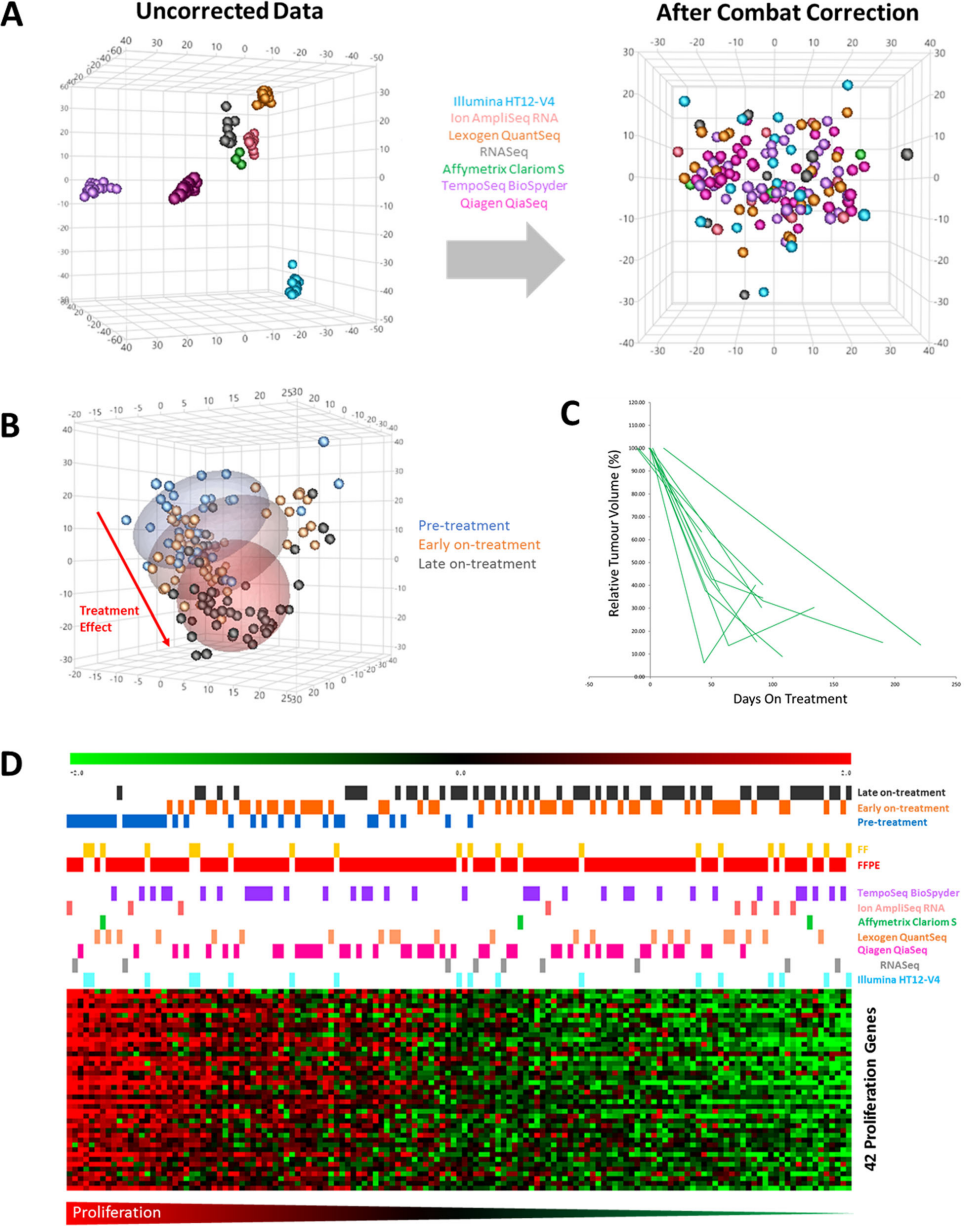
Robust gene expression measurement across platforms following batch correction. Correction of systematic platform bias and integration of data from fresh frozen and FFPE tissues. **a** 3D multi-dimensional scaling (MDS) before (left) and after (right) batch correction of 6844 common genes. Samples coloured by platform and shapes indicates time point. **b** MDS plot of the batch corrected data with samples coloured by time-point clearly demonstrates a consistent treatment effect seen across sequential patient-matched samples. **c** Ultrasound measurements of the eleven breast tumours which relate to the sequential patient-matched samples indicating consistent reductions in tumour volume over time across the patients. **d** Ranking patient samples by the expression of 42 common proliferation genes (listed in Additional file 2: Table S2) illustrates consistent changes resulting from endocrine therapy, which appears to be independent from profiling platform. Pre-treatment samples tend to have relatively high proliferation, whilst as expected early, and particularly late on-treatment samples have lower proliferation. Heatmap colours are Red = High, Green = low

Looking at the samples more closely by multi-dimensional scaling it is clear that whilst they cluster by platform before batch correction (Fig. 3a), afterwards they do not (Fig. 3b) and more importantly, instead they cluster by time point (Fig. 3c). Pre-treatment samples are most clearly separated from late on-treatment samples, with early on treatment samples in-between, as would be expected.

For further confirmation of the validity of the batch-corrected data, we ranked samples by expression of 42 proliferation genes, previously reported by us [12] that change with endocrine therapy (list of gene provided in Additional file 2: Table S2). Molecular changes in the tumours reflect the ultrasound measurements across the eleven breast tumours, concordant with consistent reductions in tumour volume over time across the patients (Fig. 3c). Ranked by proliferation genes the samples are ordered by time point, consistent with our previous results [12], and not by platform or preservation method (Fig. 3b). These results suggest that comparable gene expression profiles can be generated across the platforms using FFPE material and FFPE is a reliable alternative to FF (Fig. 3d).

## Discussion

Overall we find that gene expression data from the newer technologies is largely concordant with that from the more established methods. The newer 3′ sequencing approaches from Lexogen and Qiagen appear highly reliable and cost effective for old FFPE samples, this potentially allows valuable data to be generated from clinical samples that would not have been previously possible. The TempO-Seq method [14] from BioSpyder is an interesting approach as you can analyse expression without pre-amplification directly from a micro-dissected area of interest taken from a single FFPE section, maximizing utilization of precious or limited samples. Full RNAseq analysis is often considered the gold standard, however when tissue samples are particularly small or there is a desire to perform a range of assays or multi-omic approaches, the newer targeted sequencing approaches with many fold smaller input requirements may be a much more attractive proposition. A number of previous studies have conducted comparisons of the same samples generated from fresh and archived tissues [18, 19]. The numbers of detected genes from FFPE samples has previously been shown to be lower than from fresh tissue [19], however protocols have continued to improve [10]. It is important to remember that in all pairwise tissue comparisons where RNA is extracted separately that they cannot represent exactly the same material and are only ever adjacent, leading to inevitable potential minor variations in tissue composition. Despite this, the well-established Combat method for batch correction [17] was again found to perform well to integrate data from different sample types or technologies, this approach has been found to be superior in a many of previous studies [20].

A general finding of most platform comparison approaches is that although the correlation values between different microarray or sequencing approaches may be poor to moderate, which may relate to differences in dynamic range of the technologies, there is generally very high concordance when considering differentially expressed genes [3, 6, 21]. A comprehensive study of TCGA data found only 1.2% of genes were inconsistent by fold change [21]. A wider issue with transcriptomic studies that there is no optimal analysis pipeline for every single analysis [22].

This single study perhaps considers the widest range of gene expression technologies using FF and FFPE tissues published to date, but we acknowledge that this study documents a translational research group’s experiences, rather than being a definitive, comparison study. Not every sample was tested on every platform and some leading technologies remain to be tested, including Agilent, TaqMan and Fluidigm - due to local availability and opportunities.

## Conclusion

This study highlights the relative merits and limitations of a range of new and established gene expression profiling platforms and demonstrates that transcriptomic data from FFPE archival samples can be reliably integrated with data from FF samples, even if different measurement platforms are used. Ultimately, the choice of technology will depend upon the required resolution and coverage, throughput, sample quality, availability and budget.

## Supplementary information

**Additional file 1 : Table S1.** Table demonstrating the directly overlapping samples across the nine gene expression platforms coloured by sample type, Pink = FFPE, yellow = fresh frozen.

**Additional file 2 : Table S2.** List of the 42 proliferation-related genes showing reduction on endocrine treatment [12].

## Data Availability

All data is publicly available from NCBI GEO (www.ncbi.nlm.nih.gov/geo/) under super-series accession GSE130645.

## Abbreviations

FF: fresh frozen
FFPE: formalin-fixed paraffin embedded
RNA: Ribonucleic acid
