# Supplementary material for: Unlocking the transcriptomic potential of formalin-fixed paraffin embedded clinical tissues: comparison of gene expression profiling approaches
Source: BMC Bioinformatics. 2020 Jan 28;21:30. doi: 10.1186/s12859-020-3365-5 (PMC6988223; doi:10.1186/s12859-020-3365-5)

Supplementary Table 1

Supplementary Table 1

Table demonstrating the directly overlapping samples across the nine gene expression platforms coloured by sample type, Pink=FFPE, yellow=fresh frozen.

Gene expression platforms		P1-1	P1-2	P1-3	P2-1	P2-2	P2-3	P3-1	P3-2	P3-3	P4-1	P4-2	P4-3	P5-1	P5-2	P5-3	P6-1	P6-2	P6-3	P6-4	P7-1	P7-2	P7-3	P8-1	P8-2	P8-3	P9-1	P9-2	P9-3	P10-1	P10-2	P10-3	P11-1	P11-3
3’ RNA sequencing	Lexogen Quantseq																																	
	Qiagen Qiaseq UPX 3’																																	
Targeted sequencing	BioSpyder TempoSeq																																	
	Ion AmpliSeq																																	
Targeted probe assay	Nanostring																																	
Traditional / newer microarrays	Affymetrix Clariom S																																	
	Affymetrix U133A																																	
	Illumina HT12-V4																																	
Full RNA sequencing	RNAseq																																	

FFPE

Fresh Frozen

Graphical Abstract

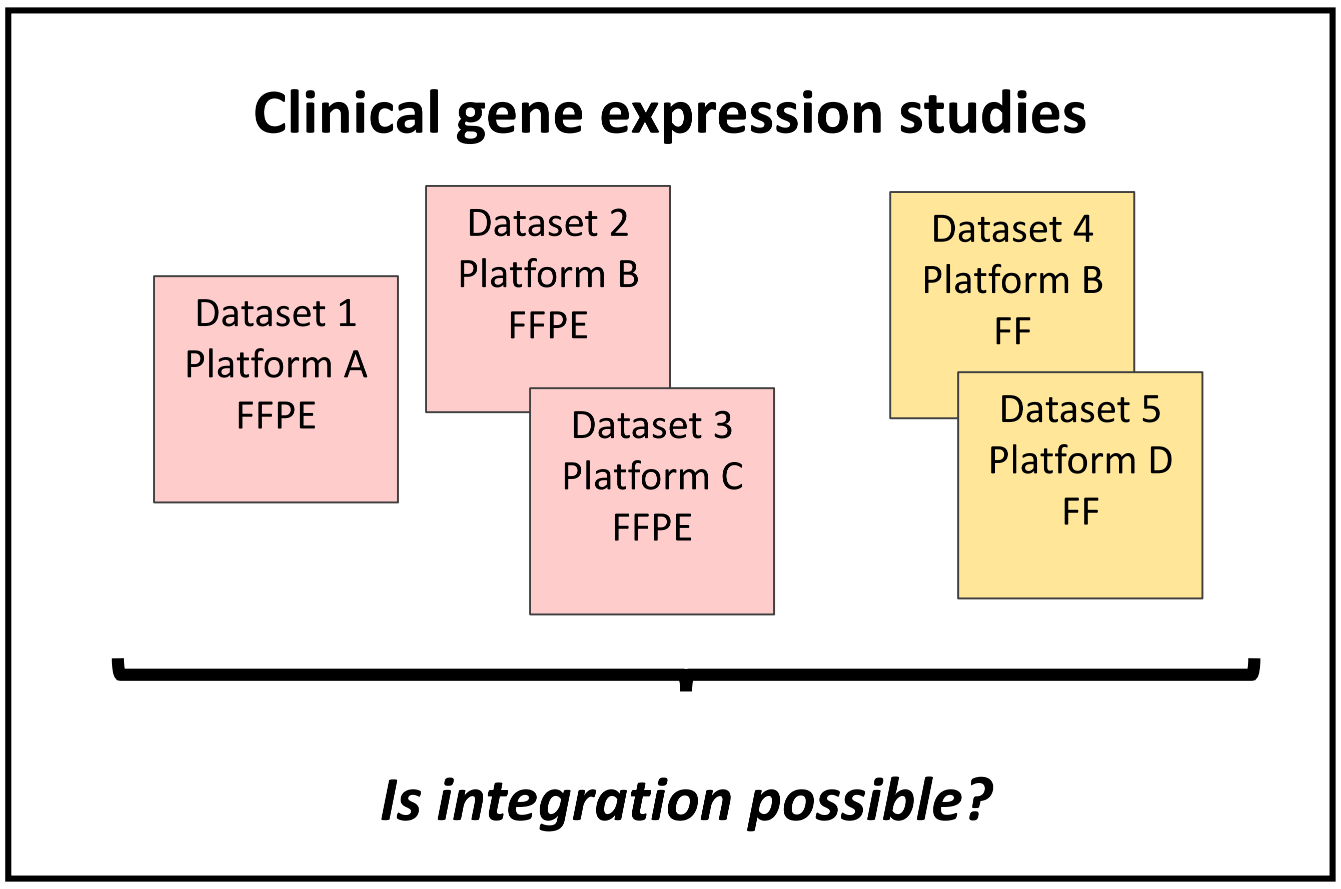

Supplement: Supplementary file 1 — Additional file 1 : Table S1. Table demonstrating the directly overlapping samples across the nine gene expression platforms coloured by sample type, Pink = FFPE, yellow = fresh frozen. [file 12859_2020_3365_MOESM1_ESM.pdf]
