# Supplementary material for: Unlocking the transcriptomic potential of formalin-fixed paraffin embedded clinical tissues: comparison of gene expression profiling approaches
Source: BMC Bioinformatics. 2020 Jan 28;21:30. doi: 10.1186/s12859-020-3365-5 (PMC6988223; doi:10.1186/s12859-020-3365-5)
Supplement: Supplementary file 2 — Additional file 2 : Table S2. List of the 42 proliferation-related genes showing reduction on endocrine treatment [12]. [file 12859_2020_3365_MOESM2_ESM.pdf]

Supplementary Table 2

ENSM Gene ID	Gene Symbol	Gene Name
ENSG00000073111	MCM2	minichromosome maintenance complex component 2(MCM2)
ENSG00000138175	ARL3	ADP ribosylation factor like GTPase 3(ARL3)
ENSG00000111666	CHPT1	choline phosphotransferase 1(CHPT1)
ENSG00000167325	RRM1	ribonucleotide reductase catalytic subunit M1(RRM1)
ENSG00000154473	BUB3	BUB3, mitotic checkpoint protein(BUB3)
ENSG00000117724	CENPF	centromere protein F(CENPF)
ENSG00000066279	ASPM	abnormal spindle microtubule assembly(ASPM)
ENSG00000134057	CCNB1	cyclin B1(CCNB1)
ENSG00000170312	CDK1	cyclin dependent kinase 1(CDK1)
ENSG00000056736	IL17RB	interleukin 17 receptor B(IL17RB)
ENSG00000122952	ZWINT	ZW10 interacting kinetochore protein(ZWINT)
ENSG00000138346	DNA2	DNA replication helicase/nuclease 2(DNA2)
ENSG00000111602	TIMELESS	timeless circadian clock(TIMELESS)
ENSG00000197061	hist1h4c	histone cluster 1 H4 family member c(HIST1H4C)
ENSG00000088325	TPX2	TPX2, microtubule nucleation factor(TPX2)
ENSG00000131153	GINS2	GINS complex subunit 2(GINS2)
ENSG00000126803	HSPA2	heat shock protein family A (Hsp70) member 2(HSPA2)
ENSG00000110092	CCND1	cyclin D1(CCND1)
ENSG00000102384	CENPI	centromere protein I(CENPI)
ENSG00000132646	PCNA	proliferating cell nuclear antigen(PCNA)
ENSG00000164104	HMGB2	high mobility group box 2(HMGB2)
ENSG00000140443	IGF1R	insulin like growth factor 1 receptor(IGF1R)
ENSG00000137804	NUSAP1	nucleolar and spindle associated protein 1(NUSAP1)
ENSG00000106462	EZH2	enhancer of zeste 2 polycomb repressive complex 2 subunit(EZH2)
ENSG00000104738	MCM4	minichromosome maintenance complex component 4(MCM4)
ENSG00000145386	CCNA2	cyclin A2(CCNA2)
ENSG00000198087	CD2AP	CD2 associated protein(CD2AP)
ENSG00000101773	MIR4741	microRNA 4741(MIR4741)
ENSG00000131747	TOP2A	topoisomerase (DNA) II alpha(TOP2A)
ENSG00000188486	H2AFX	H2A histone family member X(H2AFX)
ENSG00000164032	H2AFZ	H2A histone family member Z(H2AFZ)
ENSG00000138180	CEP55	centrosomal protein 55(CEP55)
ENSG00000136518	ACTL6A	actin like 6A(ACTL6A)
ENSG00000173473	SMARCC1	SWI/SNF related, matrix associated, actin dependent regulator of chromatin subfamily c member 1(SMARCC1)
ENSG00000126787	DLGAP5	DLG associated protein 5(DLGAP5)
ENSG00000109084	TMEM97	transmembrane protein 97(TMEM97)
ENSG00000163918	RFC4	replication factor C subunit 4(RFC4)
ENSG00000076003	MCM6	minichromosome maintenance complex component 6(MCM6)
ENSG00000109805	NCAPG	non-SMC condensin I complex subunit G(NCAPG)
ENSG00000196074	SYCP2	synaptonemal complex protein 2(SYCP2)
ENSG00000198901	PRC1	protein regulator of cytokinesis 1(PRC1)
ENSG00000175063	UBE2C	ubiquitin conjugating enzyme E2 C(UBE2C)
